# *TLR3* gene polymorphisms in cancer: a systematic review and meta-analysis

**DOI:** 10.1186/s40880-015-0020-z

**Published:** 2015-06-10

**Authors:** Ben-Gang Wang, De-Hui Yi, Yong-Feng Liu

**Affiliations:** Department 1 of General Surgery, the First Affiliated Hospital of China Medical University, Shenyang, Liaoning 110001 Peoples Republic of China

**Keywords:** The Toll-like receptor 3 (*TLR3*) gene, Single nucleotide polymorphism, Cancer risk, Meta-analysis, Systematic review

## Abstract

**Introduction:**

Recent studies examining the association of Toll-like receptor 3 (*TLR3*) gene polymorphisms with the risk of developing various types of cancer have reported conflicting results. Clarifying this association could advance our knowledge of the influence of *TLR3* single nucleotide polymorphisms (SNPs) on cancer risk.

**Methods:**

We systematically reviewed studies that focused on a collection of 12 SNPs located in the *TLR3* gene and the details by which these SNPs influenced cancer risk. Additionally, 14 case-control studies comprising a total of 7997 cases of cancer and 8699 controls were included in a meta-analysis of 4 highly studied SNPs (rs3775290, rs3775291, rs3775292, and rs5743312).

**Results:**

The variant *TLR3* genotype rs5743312 (C9948T, intron 3, C > T) was significantly associated with an increased cancer risk as compared with the wild-type allele (odds ratio [OR] = 1.11, 95 % confidence interval [CI] = 1.00–1.24, *P* = 0.047). No such association was observed with other *TLR3* SNPs. In the stratified analysis, the rs3775290 (C13766T, C > T) variant genotype was found to be significantly associated with an increased cancer risk in Asian populations. Additionally, the rs3775291 (G13909A, G > A) variant genotype was significantly associated with an increased cancer risk in Asians, subgroup with hospital-based controls, and subgroup with a small sample size.

**Conclusion:**

After data integration, our findings suggest that the *TLR3* rs5743312 polymorphism may contribute to an increased cancer risk.

## Background

Toll-like receptors (TLRs) are members of a membrane receptor protein family that recognize the antigenic determinants of viruses, bacteria, protozoa, and fungi, and are thus associated with immunity. There are two pathways associated with the immune deficiencies that lead to disease: the MyD88-IRAK4 pathway, involving all TLR family proteins except TLR3, and the TLR3-Unc93b-TRIF-TRAF3 pathway [1, 2]. Therefore, TLR3 can be considered a unique protein in the TLR family, and it has been further implicated in the development of tumors resulting from an activated immune system.

*TLR3* single nucleotide polymorphisms (SNPs) could possibly affect cancer susceptibility and could therefore serve as potential biomarkers to evaluate cancer risk [3]. Thus, *TLR3* polymorphisms that are associated with both heredity and environmental factors may be critical in bridging the relationship between genetic factors and external environmental conditions. *TLR3* SNPs were originally identified by *He* et al. [4] in 2007. Since then, several studies have focused on examining the association of *TLR3* SNPs with cancer risk. However, the first studied SNP site included 10 SNPs covering the entire *TLR3* gene, and because each of these SNPs had several reported names, confusion arose in the literature. Furthermore, published results of the *TLR3* gene have been conflicting. The *TLR3* SNPs that exhibit the most variability are rs3775290 and rs3775291; however, their relationship with cancer risk remains unclear. For instance, although the majority of studies have reported that the rs3775290 variant T allele was associated with an increased cancer risk (odds ratio [OR] > 1), four studies testing this conclusion did not reach statistical significance (*P* > 0.05) [5–8] and only one additional study produced significant results (*P* < 0.05) [9]. Furthermore, another study reported the opposite result that the variant allele was associated with a decreased cancer risk (OR = 0.88) [4]. Thus, a comprehensive analysis integrating all studies on *TLR3* SNPs and cancer risk is needed. In addition, no systematic review or meta-analysis for the *TLR3* gene polymorphisms has been performed.

Here, we systematically reviewed published data and comprehensively analyzed and integrated all published studies on the relationship between *TLR3* SNPs and cancer risk. We also contacted the authors of these studies to obtain any data that was omitted from published articles so as to enhance our comprehension of the details surrounding these SNPs. We included a meta-analysis for hotspot SNPs (rs3775290, rs3775291, rs3775292, and rs5743312) that have been described in at least three published studies to enhance the comprehensiveness of our assessment into the association between *TLR3* SNPs and cancer risk.

## Methods

### Publication search

A systematic literature search was performed on the association between the *TLR3* rs3775290, rs3775291, rs3775292, rs5743312 polymorphisms and cancer risk. Our final search concluded with literature published on or before October 5th, 2014. Two independent researchers (Ben-Gang Wang and De-Hui Yi) searched the PubMed, Chinese National Knowledge Infrastructure (CNKI), and Web of Science databases for the following keywords: “*TLR3*,” “cancer/carcinoma/tumor/neoplasm,” and “polymorphism”. The inclusion criteria were as follows: (1) case-control studies, (2) studies evaluating the association between *TLR3* polymorphisms and cancer risk, and (3) the SNP was reported in at least 3 publications. The major exclusion criteria were as follows: (1) studies that presented duplicate data, (2) studies that included only cancer patients (i.e., no healthy controls), and (3) studies that investigated benign diseases compared with controls.

### Data extraction

Two independent researchers (Ben-Gang Wang and De-Hui Yi) each extracted all data that were considered to be relevant, and in cases of inconsistent selection, a third author (Yong-Feng Liu) participated in data selection. In this way, a consensus was reached on which studies should be included for analysis. For each study, the following items were collected: first author’s name, publication year, country of origin, ethnicity, cancer type, source of control groups (population- or hospital-based), genotyping method, total numbers of cases and controls, and genotype distributions in cases and controls. When we considered the published data to be insufficient for our analyses, we contacted the authors to obtain the original data. Thus, the data included in this review were obtained from both published and unpublished studies.

### Statistical analyses

The Chi-square test was used to determine the Hardy-Weinberg equilibrium (HWE) for the genotype frequencies of different *TLR3* polymorphisms; a result of *P* < 0.05 was considered to indicate significant disequilibrium. The strength of the association between *TLR3* polymorphisms and cancer risk was calculated using odds ratios (ORs) with 95 % confidence intervals (CIs). The heterogeneity between different studies was calculated using the Cochran’s *Q* test and quantified by the *I*^*2*^ (a significance level of *P* < 0.10). When heterogeneity did not exist, a fixed-effect model based on the Mantel-Haenszel method was used to assess the pooled OR of each study [10]. When heterogeneity did exist, a random-effect model based on the method developed by DerSimonian and Laird was employed [11]. Five comparison models were evaluated: heterozygote comparison (M1: Aa vs. AA), homozygote comparison (M2: aa vs. AA), dominant model (M3: Aa + aa vs. AA), recessive model (M4: aa vs. AA + Aa), and allelic model (M5: a vs. A). “A” indicates wild allele, and “a” indicates variant allele. OR_1_ and OR_2_ were calculated for the genotypes aa vs. AA (M2) and Aa vs. AA (M1), and were used to determine the most appropriate genetic model. According to the reference [12], a recessive model is recommended in cases where OR_1_ ≠ 1 and OR_2_ = 1, whereas a dominant model is suggested for cases in which OR_1_ = OR_2_ ≠ 1, and a codominant model is indicated if OR_1_ > OR_2_ > 1 or OR_1_ < OR_2_ < 1.

For studies with sufficient patients, we also performed stratification analyses on cancer type, ethnicity (Asian or Caucasian), sources of controls (population- or hospital-based study design), and sample size (total samples ≥1000 [large sample size] or <1000 [small sample size]). The Begg’s rank correlation and the Egger’s linear regression tests were used to evaluate publication bias [13, 14]. A value of *P* < 0.10 was considered statistically significant. All analyses were performed using STATA software, version 11.0 (STATA Corp., College Station, TX, USA).

## Results

### Characteristics of the included studies

Searches of the PubMed, CNKI, and Web of Science databases using different combinations of our keywords yielded a total of 155 records (after duplicates were removed). We excluded 50 studies based on the information presented in the title or abstract (17 were irrelevant articles, 16 were functional studies rather than polymorphism studies, and 17 were review articles) and 91 studies based on the information presented in the text (5 were not case-control studies, 62 were not about *TLR3* gene polymorphisms, and 24 were not relevant to cancer). Thus, a total of 14 case-control studies that met our inclusion criteria were included in our systematic review and final meta-analysis, which consisted of 7997 cancer patients and 8699 cancer-free controls [4–9, 15–22] (Table 1).

**Table 1:** The studies included in this systematic review for the association between Toll-like receptor 3 (*TLR3*) single nucleotide polymorphisms (SNPs) and the risk of developing cancer

Publication year	Study	Country	Sample size		Source of controls	Genotyping method	Matched factors	Adjusted factors
			Cases	Controls				
2007	He *et al.* [4]	China	434	512	Population-based	Sequencing	Sex and age matched	None
2009	Etokebe *et al.* [8]	Croatia	130	101	Population-based	qPCR	All females, age not matched	None
2010	Lei *et al.* [15]	China	981	1221	Population-based	SNP Stream	All females, age matched	Age and BMI
2011	Pandey *et al.* [7]	India	200	200	Not mentioned, only healthy controls	PCR-RFLP	All females, age matched	Age
	Gast *et al.* [16]	Germany	763	736	Hospital-based	Two multiplex PCR	Sex not mentioned, age not matched	None
2012	Mandal *et al.* [6]	India	195	250	Hospital-based	PCR-RFLP	All males, age matched	
	Slattery *et al*.[17]	USA	2309	2915	Population-based	Multiplexed bead array	Sex and age matched	None
2013	Singh *et al.* [5]	India	200	200	Hospital-based	PCR-RFLP	Sex and age matched	Age, sex, and smoking
	Li *et al.* [9]	China	466	482	Population-based	PCR-RFLP	Sex and age matched	Sex and age
	Resler *et al.* [18]	USA	840	800	Population-based	Chip	All females, age matched	Age
	Zeljic *et al.* [19]	Yugoslavia	93	104	Not mentioned, only healthy controls	qPCR	Sex and age matched	Sex, age, smoking, and alcohol consumption
	Yeyeodu *et al.* [20]	USA	102	72	Hospital-based	SNP Stream	All females, age not mentioned	None
	Moumad *et al.* [21]	Germany	472	362	Hospital-based	KASPar	Sex and age matched	Sex and age
2014	Zidi *et al.* [22]	Tunisia	130	200	Hospital-based	PCR-RFLP	All females, age not matched	None

qPCR, quantitative polymerase chain reaction; PCR-RFLP, polymerase chain reaction-restriction fragment length polymorphism; KASPar, KASPar SNP genotyping system; BMI, body mass index.

Of the 14 enrolled studies, all but 4 were matched by age; 6 were sex-matched, 1 did not mention the sex-matching, and 7 that examined either breast, cervical, or prostate cancer did not need to match for sex. Six studies investigated Asians, 7 investigated Caucasians, and 1 was conducted in Africa. The controls were hospital-based in 6 studies, population-based in 6 studies, and not mentioned in 2 studies. Genotyping methods included polymerase chain reaction-restriction fragment length polymorphism (PCR-RFLP, 5 studies), quantitative polymerase chain reaction (qPCR, 2 studies), Chip (1 study), multiplexed bead array (1 study), two multiplex PCR (1 study), the KASPar SNP genotyping system (1 study), SNP Stream (2 studies), and sequencing (1 study). Eight studies checked genotypes for quality control [4–6, 9, 16–18, 21]. The genotype distribution of controls was consistent with the Hardy-Weinberg equilibrium model in all but four studies [9, 15, 17, 22].

In these 14 studies, 12 SNPs were reported. Table 2 lists the SNP locations, studied tumor types, and any associations between *TLR3* SNPs and cancer risk. In summary, there are 10 types of cancer that have been evaluated in relation to *TLR3* SNPs. The majority of the 12 SNPs located within an intronic region of the *TLR3* gene, except for 2 that were found in the 3′ and 5′ regions of the gene and another 2 that located within exons. Only 4 SNPs (rs3775290, rs3775291, rs3775292, and rs5743312) were reported in at least 3 studies. For this reason, we defined them as hotspot SNPs which covered all 10 cancer types included in this study (Table 3).

**Table 2:** The associations between *TLR3* polymorphisms and cancer risk in the 14 selected studies

*TLR3* SNP	Location	Association(s) with tumor(s)
rs10025405	3′-near gene	Breast cancer, associated [20]
rs11721827	Intron 1	Nasopharyngeal carcinoma, associated [4]
		Colorectal cancer, associated [17]
rs11730143	Intron 1	Melanoma, not associated [16]
rs13126816	Intron 1	Melanoma, not associated [16]
rs3775290	Exon 4	Nasopharyngeal carcinoma, not associated [4]
		Bladder cancer, not associated [5]
		Prostate cancer, not associated [6]
		Cervical cancer, not associated [7]
		Breast cancer, not associated [8]
		Cervical cancer, associated [22]
rs3775291	Exon 4, L412F	Nasopharyngeal carcinoma, not associated [4]
		Hepatocellular carcinoma, associated [9]
		Breast cancer, not associated [15]
		Melanoma, not associated [16]
		Colorectal cancer, not associated [17]
		Breast cancer, not associated [18]
		Oral cancer, associated [19]
		Nasopharyngeal carcinoma, associated [21]
rs3775292	Intron 3	Melanoma, not associated [16]
		Colorectal cancer, associated [17]
		Breast cancer, not associated [18]
rs5743305	5′-near gene	Hepatocellular carcinoma, not associated [9]
		Colorectal cancer, not associated [17]
rs5743312	Intron 3	Nasopharyngeal carcinoma, not associated [4]
		Melanoma, not associated [16]
		Oral cancer, associated with survival [19]
rs7657186	Intron 1	Melanoma, not associated [16]
		Breast cancer, not associated [20]
rs7668666	Intron 3	Melanoma, not associated [16]

**Table 3:** Characteristics of the 14 studies included for this meta-analysis

Study	Ethnicity	Cancer type	Sample size		Cases			Controls			*P* of HWE
			Case	Control	wt/wt	wt/var	var/var	wt/wt	wt/var	var/var	
rs3775290 (C13766T, C > T)											
He *et al*. [4]	Asian	Nasopharyngeal carcinoma	395	371	565^*^		225^*^	510^*^		232^a^	
Singh *et al*. [5]	Asian	Bladder cancer	200	200	106	81	13	122	71	7	0.391
Mandal *et al*. [6]	Asian	Prostate cancer	195	250	115	68	12	157	84	9	0.585
Pandey *et al*. [7]	Asian	Cervical cancer	200	200	91	98	11	110	81	9	0.217
Etokebe *et al*. [8]	Caucasian	Breast cancer	130	101	58	56	14	46	42	8	0.713
Zidi *et al*. [22]	Caucasian	Cervical cancer	130	200	69	48	13	76	106	18	0.026
rs3775291 (G13909A, L412F, G > A)											
He *et al*. [4]	Asian	Nasopharyngeal carcinoma	333	287	405^*^		261^*^	359^a^		215^a^	
Li *et al*. [9]	Asian	Hepatocellular carcinoma	466	482	192	222	52	256	203	23	0.030
Lei *et al*. [15]	Asian	Breast cancer	981	1221	447	431	103	594	500	127	0.155
Gast *et al*. [16]	Caucasian	Skin malignant melanoma	732	668	379	291	62	332	284	52	0.415
Slattery *et al*. [17]	Caucasian	Colon cancer	1554	1955	748	653	153	947	817	191	0.446
		Rectal cancer	754	959	**363**	**332**	59	**478**	**381**	100	0.066
Resler *et al*. [18]	Caucasian	Breast cancer	840	801	427	348	65	418	318	65	0.679
Zeljic *et al*. [19]	Caucasian	Oral squamous cell carcinomas	93	104	39	39	15	43	53	8	0.128
Moumad *et al*. [21]	African	Nasopharyngeal carcinoma	472	362	289	170	13	252	96	14	0.210
rs3775292 (C/G, intron 3, C > G)											
Gast *et al*. [16]	Caucasian	Skin malignant melanoma	730	668	464	233	33	413	228	27	0.521
Slattery *et al*. [17]	Caucasian	Colon cancer	1554	1956	**990**	**507**	57	**1253**	**601**	102	0.008
		Rectal cancer	754	959	494	**222**	**38**	586	**335**	**38**	0.247
Resler *et al*. [18]	Caucasian	Breast cancer	840	800	522	284	34	509	262	29	0.507
rs5743312 (C9948T, intron 3, C > T)											
He *et al*. [4]	Asian	Nasopharyngeal carcinoma	405	200	640^*^		170^a^	316^*^		84^a^	
Lei *et al*. [15]	Asian	Breast cancer	996	1245	582	348	66	770	433	42	0.044
Gast *et al*. [16]	Caucasian	Skin malignant melanoma	716	657	500	200	16	453	193	11	0.060
Zeljic *et al*. [19]	Caucasian	Oral squamous cell carcinomas	93	104	69	21	3	78	24	2	0.923

wt/wt, wild type/wild type, indicating wild genotype; wt/var, wild type/variant type, indicating heterozygote; var/var, variant type/variant type, indicating variant genotype; HWE, Hardy-Weinberg equilibrium. ^*^Only the data of allelic model were available, and thus the studies were analyzed only when the allelic model was used. The bold means the genotype information of the rs3775291 and rs3775292 SNPs that readers could not found in [17], and these data were kindly provided by the contacted authors.

### Unpublished data obtained from the original authors

Slattery *et al*. [17] detected *TLR3* rs3775291 and rs3775292 SNPs in colorectal cancer patients and showed only the minimum allele frequency for these SNPs. We contacted the authors of this study to obtain the genotype information of these two SNPs that were found in the case and control groups, and we were especially interested in the results when dividing patients into colon cancer and rectal cancer groups. These results are presented in bold in Table 3.

### Quantitative synthesis

**Fig. 1:**
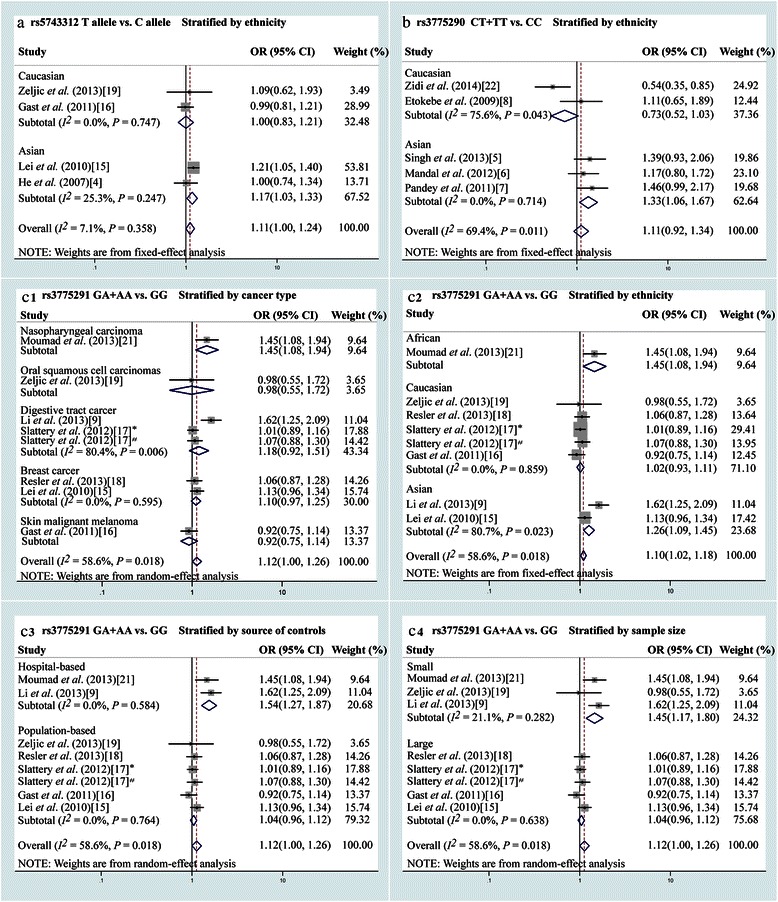
Forest plot of the odds ratios (ORs) for the association of Toll-like receptor 3 (*TLR3*) single nucleotide polymorphisms (SNPs) with cancer risk. **a**, the association of the *TLR3* rs5743312 SNP with cancer risk when stratified by ethnicity (allelic model). **b**, the association of the *TLR3* rs3775290 SNP with cancer risk when stratified by ethnicity (dominant model). **c**, The association of *TLR3* rs3775291 SNP with cancer risk (dominant model) when stratified by cancer type (c1), ethnicity (c2), source of controls (c3), and sample size (c4). *The cancer type was colon cancer; ^#^the cancer type was rectal cancer

The variant T allele of the rs5743312 SNP was significantly associated with an increased risk of cancer when compared with the wild C allele (OR = 1.11, 95 % CI = 1.00–1.24, *P* = 0.047) (Table 4, Fig. 1a). The OR_1_ and OR_2_ values of this rs5743312 SNP were 1.88 (*P* < 0.001) and 1.02 (*P* = 0.832), respectively. Thus, a codominant model (M2) was the most appropriate choice for rs5743312. Regardless of the genetic model, no other *TLR3* SNPs (rs3775290, rs3775291, and rs3775292) were found to be associated with cancer risks.

**Table 4:** Pooled ORs and 95 % CIs of *TLR3* polymorphisms in this meta-analysis

SNP	Number of studies	M1			M2			M3			M4			Number of studies	M5		
		OR (95 % CI)	*P*	*I* ^*2*^ (%)	OR (95 % CI)	*P*	I^2^ (%)	OR (95 % CI)	*P*	I^2^ (%)	OR (95 % CI)	*P*	I^2^ (%)		OR (95 % CI)	*P*	I^2^ (%)
rs3775290 (C13766T, C > T)	5	1.04	0.854^*^	70.0	1.38	0.110	0.0	1.09	0.640^*^	69.4	1.41	0.082	0.0	6	1.06	0.550	59.3
		(0.72-1.49)			(0.93-2.06)			(0.77-1.53)			(0.96-2.08)				(0.87-1.30)		
rs3775291 (G13909A, L412F, G > A)	8	1.12	0.056^*^	53.9	1.13	0.335^*^	66.5	1.12	0.054^*^	58.6	1.08	0.507^*^	65.2	9	1.09	0.064^*^	59.5
		(1.00-1.26)			(0.88-1.45)			(1.00-1.26)			(0.86-1.37)				(1.00-1.19)		
rs3775292 (C/G, intron 3, C > G)	4	0.96	0.554^*^	54.5	0.93	0.500	34.7	0.97	0.476	23.1	0.94	0.554	49.9	4	0.97	0.416	0.0
		(0.83-1.11)			(0.75-1.15)			(0.89-1.06)			(0.76-1.16)				(0.90-1.05)		
rs5743312 (C9948T, intron 3, C > T)	3	1.02	0.832	0.0	**1.88**	**<0.001**	0.0	1.08	0.267	0.0	**1.86**	**<0.001**	0.0	4	**1.11**	**0.047**	7.1
		(0.88-1.17)			**(1.33-2.67)**			(0.94-1.23)			**(1.32-2.63)**				**(1.00-1.24)**		

OR, odds ratio; CI, confidence interval. ^*^The heterogeneity exists; a random-effect model based on the DerSimonian and Laird method or a fixed-effect model based on the Mantel-Haenszel method was used. M1: Aa vs. AA, heterozygote comparison; M2: aa vs. AA, homozygote comparison; M3: Aa + aa vs. AA, dominant model; M4: aa vs. AA + Aa, recessive model; M5: a vs. A, allelic model (A indicates wild allele, a indicates variant allele). The bold means the significant results.

In the stratified analysis, the rs3775290 variant genotype was significantly associated with an increased cancer risk in Asian populations in M1 (CT vs. CC: OR = 1.28, 95 % CI = 1.02–1.62, *P* = 0.036), M2 (TT vs. CC: OR = 1.79, 95 % CI = 1.05–3.05, *P* = 0.032), and M3 models (CT + TT vs. CC: OR = 1.33, 95 % CI = 1.06–1.67, *P* = 0.013) (Table 5, Fig. 1b). For the rs3775291 SNP, both the GA heterozygote and the GA + AA genotypes were consistently associated with an increased risk of nasopharyngeal cancer (GA vs. GG: OR = 1.54, 95 % CI = 1.14–2.09, *P* = 0.005; GA + AA vs. GG: OR = 1.45, 95 % CI = 1.09–1.94, *P* = 0.012) (Table 5, Fig. 1c1). When the data were stratified by ethnicity, the GA heterozygote was significantly associated with an increased cancer risk in the Asian subgroup (GA vs. GG: OR = 1.27, 95 % CI = 1.00–1.60, *P* = 0.048) (Table 5, Fig. 1c2). When the data were stratified by the source of controls, the hospital-based subgroup showed that the variant allele was significantly associated with an increased cancer risk (GA vs. GG: OR = 1.50, 95 % CI = 1.23–1.83, *P* < 0.001; GA + AA vs. GG: OR = 1.54, 95 % CI = 1.27–1.87, *P* < 0.001; A vs. G: OR = 1.43, 95 % CI = 1.23–1.67, *P* < 0.001) (Table 5, Fig. 1c3). Finally, when the data were stratified by sample size, the small sample size subgroup showed that the variant genotype was significantly associated with an increased cancer risk (AA vs. GG: OR = 2.77, 95 % CI = 1.75–4.39, *P* < 0.001; AA vs. GA + GG: OR = 2.46, 95 % CI = 1.58–3.83, *P* < 0.001) (Table 5, Fig. 1c4).

**Table 5:** Pooled ORs and 95 % CIs of *TLR3* polymorphisms in the stratified analysis

Stratification	Number of studies	M1			M2			M3			M4			Number of studies	M5		
		OR (95 % CI)	*P*	*I* ^2^ (%)	OR (95 % CI)	*P*	*I* ^2 ^(%)	OR (95 % CI)	*P*	*I* ^2 ^(%)	OR (95 % CI)	*P*	*I* ^2 ^(%)		OR (95 % CI)	*P*	*I* ^2 ^(%)
rs3775290 (C13766T, C > T)																	
Ethnicity																	
Asian	3	**1.28**	**0.036**	0	**1.79**	**0.032**	0	**1.33**	**0.013**	0	1.61	0.08	0	4	1.14	0.25^a^	58.2
		**(1.02-1.62)**			**(1.05-3.05)**			**(1.06-1.67)**			(0.95-2.71)				(0.91-1.43)		
Caucasian	2	0.72	0.372	75.5	1.00	0.996	0	0.76	0.45^a^	75.6	1.35	0.52	0	2	0.89	0.60^a^	63.6
		(0.34-1.49)			(0.55-1.82)			(0.38-1.54)			(0.54-3.36)				(0.58-1.38)		
Cancer type																	
Genital system neoplasms	4	0.97	0.892	75	1.26	0.3	0	1.04	0.73^a^	73.9	1.33	0.2	0		1.07	0.64^a^	58.8
		(0.62-1.53)			(0.81-1.95)			(0.84-1.29)			(0.87-2.03)				(0.82-1.39)		
Source of controls																	
Hospital-based		NA			NA			NA			NA			5	1.12	0.32^a^	54.7
															(0.90-1.40)		
Population-based		NA			NA			NA			NA			1	0.88	0.23	
															(0.70-1.09)		
rs3775291 (G13909A, L412F, G > A)																	
Cancer type																	
Digestive tract cancer	3	1.16	0.132^a^	65.4	1.28	0.429	89.1	1.18	0.186	80.4	1.18	0.559^a^	87.4	3	1.14	0.288^a^	87.5
		(0.96-1.41)			(0.69-2.38)			(0.92-1.51)			(0.68-2.05)				(0.90-1.44)		
Breast cancer	2	1.11	0.118	0	1.04	0.739	0	1.10	0.148	0	0.99	0.911	0	2	1.05	0.288	0
		(0.97-1.27)			(0.70-1.55)			(0.97-1.25)			(0.79-1.23)				(0.96-1.16)		
Nasopharyngeal carcinoma	1	**1.54**	**0.005**		0.81	0.593		**1.45**	**0.012**		0.70	0.37		2	1.16	0.083	0
		**(1.14-2.09)**			(0.37-1.76)			**(1.09-1.94)**			(0.33-1.52)				(0.98-1.37)		
Ethnicity																	
Caucasian	5	1.02	0.613	0	0.98	0.78	4.6	1.02	0.732	0	0.97	0.685	40.9	5	1.00	0.94	0
		(0.94-1.12)			(0.84-1.14)			(0.93-1.11)			(0.84-1.13)				(0.93-1.07)		
Asian	2	**1.27**	**0.048** ^**a**^	54	1.76	0.272	91.2	1.33	0.105	80.7	1.55	0.334	89.5	3	1.21	0.108^a^	79.5
		**(1.00-1.60)**			(0.64-4.82)			(0.94-1.89)			(0.64-3.77)				(0.96-1.53)		
African	1	**1.23**	**0.005**		0.81	0.593		**1.45**	**0.012**		0.70	0.37		1	1.27	0.062	
		**(1.06-1.43)**			(0.37-1.76)			**(1.09-1.94)**			(0.33-1.52)				(0.99-1.63)		
Source of controls																	
Hospital-based	2	**1.50**	**<0.001**	0	1.61	0.466^a^	86.8	**1.54**	**<0.001**	0	1.37	0.616^a^	86.3	2	**1.43**	**<0.001**	32.6
		**(1.23-1.83)**			(0.45-5.84)			**(1.27-1.87)**			(0.40-4.76)				**(1.23-1.67)**		
Population-based	6	1.05	0.259	0	1.00	0.999	0	1.04	0.338	0	0.98	0.748	26.9	7	1.02	0.465^a^	0
		(0.97-1.14)			(0.87-1.15)			(0.96-1.12)			(0.86-1.12)				(0.97-1.08)		
Sample size																	
Large	5	1.05	0.218	0	0.98	0.823	0	1.04	0.328	0	0.96	0.545	0	5	1.02	0.629	0
		(0.97-1.14)			(0.86-1.13)			(0.96-1.12)			(0.84-1.10)				(0.96-1.08)		
Small	3	**1.41**	**<0.001**	45.6	1.76	0.184^a^	73.7	**1.47**	**<0.001**	21.1	1.63	0.242^a^	73.9	4	**1.30**	**<0.001**	47.9
		**(1.16-1.70)**			(0.77-4.05)			**(1.23-1.76)**			(0.72-3.65)				**(1.15-1.47)**		
rs3775292 (intron 3, C > G)																	
Cancer type																	
Colorectal cancer	2	0.93	0.609^a^	82.2	0.89	0.657^a^	68	0.93	0.464^a^	63.8	0.92	0.797^a^	78.2	2	0.94	0.233	0
		(0.69-1.25)			(0.54-1.48)			(0.76-1.13)			(0.50-1.69)				(0.86-1.04)		
Breast Cancer	1	1.06	0.601		1.14	0.607		1.07	0.535		1.12	0.656		1	1.06	0.499	
		(0.86-1.30)			(0.69-1.90)			(0.87-1.30)			(0.68-1.86)				(0.90-1.26)		
Skin malignant melanoma	1	0.91	0.411		1.09	0.753		0.93	0.503		1.12	0.659		1	0.96	0.683	
		(0.73-1.14)			(0.64-1.84)			(0.75-1.15)			(0.67-1.89)				(0.80-1.16)		
rs5743312 (C9948T, intron 3, C > T)																	
Ethnicity																	
Asian		NA			NA			NA			NA			2	**1.17**	**0.017**	25.3
															**(1.03-1.33)**		
Caucasian		NA			NA			NA			NA			2	0.99	0.995	0
															(0.83-1.21)		
Sample size																	
Large	2	1.02	0.82	0	**1.89**	**<0.001**	4	1.08	0.268	37.2	**1.87**	**0.001**	0	2	1.11	0.305^a^	61.7
		(0.88-1.17)			**(1.32-2.70)**			(0.94-1.24)			**(1.31-2.65)**				(0.99-1.24)		
Small	1	0.99	0.97		1.70	0.569		1.04	0.9		1.70	0.566		2	1.02	0.894	0
		(0.51-1.93)			(0.28-10.45)			(0.55-1.98)			(0.28-10.40)				(0.78-1.32)		

NA, not available. Other footnotes as in Table 4. The bold means the significant results.

### Heterogeneity

The heterogeneities that originated within the collection of selected studies and within each subgroup of studies are shown in Tables 4 and 5, respectively. Slight heterogeneities were found when comparing different studies. To explore the influence of individual studies on the pooled results, we analyzed the sensitivity of our methodology by removing one study at a time from the pooled analyses. No significant heterogeneity was found for any genetic model, which suggested that our results were relatively reliable.

### Publication bias

The Begg’s rank correlation and Egger’s linear regression tests were conducted to evaluate publication bias. According to the results of these tests, a slight publication bias for rs3775290 in M2 was indicated (Table 6).

**Table 6:** The results of Begg’s and Egger’s test for the publication bias

Comparison model	Begg’s test		Egger’s test	
	*Z* value	*P* value	*t* value	*P* value
rs3775290 (C13766T, C > T)				
M1	−0.98	0.327	−0.88	0.443
M2	0.98	0.327	3.08	0.054
M3	−0.98	0.327	−0.78	0.493
M4	1.47	0.142	1.7	0.188
M5	−0.19	0.851	0.87	0.432
rs3775291 (G13909A, L412F, G > A)				
M1	0.49	0.621	0.62	0.559
M2	0.99	0.322	1.06	0.329
M3	0.49	0.621	1.02	0.346
M4	0.99	0.322	1.02	0.348
M5	1.25	0.211	1.48	0.183
rs3775292 (C/G, intron 3, C > G)				
M1	−1.36	0.174	−1.14	0.371
M2	0.00	1.000	3.88	0.061
M3	0.00	1.000	−0.68	0.567
M4	0.00	1.000	3.11	0.090
M5	0.68	0.497	0.14	0.901
rs5743312 (C9948T, intron 3, C > T)				
M1	−0.52	0.602	−0.39	0.764
M2	−0.52	0.602	−0.76	0.587
M3	−0.52	0.602	0.75	0.590
M4	−0.52	0.602	−0.75	0.589
M5	0.00	1.000	−0.86	0.479

NA, not available. Other footnotes as in Table 4.

## Discussion

In this meta-analysis, we found that *TLR3* rs5743312 was associated with an increased overall risk of developing cancer. This was also true for the large sample size subgroup. *TLR3* rs3775290 and rs3775291 polymorphisms were found to be associated with increased cancer risks in the stratified analysis, whereas no association was found between *TLR3* rs3775292 and cancer risk.

The *TLR3* rs5743312 polymorphism is located in intron 3, and no previous studies have shown any association between this polymorphism and cancer risk. However, after data integration, we concluded that this SNP is the only significant site in the *TLR3* gene with respect to cancer risk. According to our meta-analysis, the remaining 3 SNPs exhibited no association with cancer risks. The subgroup analyses for the rs5743312 SNP showed the same tendency as the whole group analysis. Previous studies have suggested that intronic SNPs may exhibit specific functions, such as directing alternative splicing [23, 24]. As intronic polymorphisms have been shown to exhibit critical functions, it would be prudent to include intronic SNPs, such as rs5743312, in future studies.

**Fig. 2:**
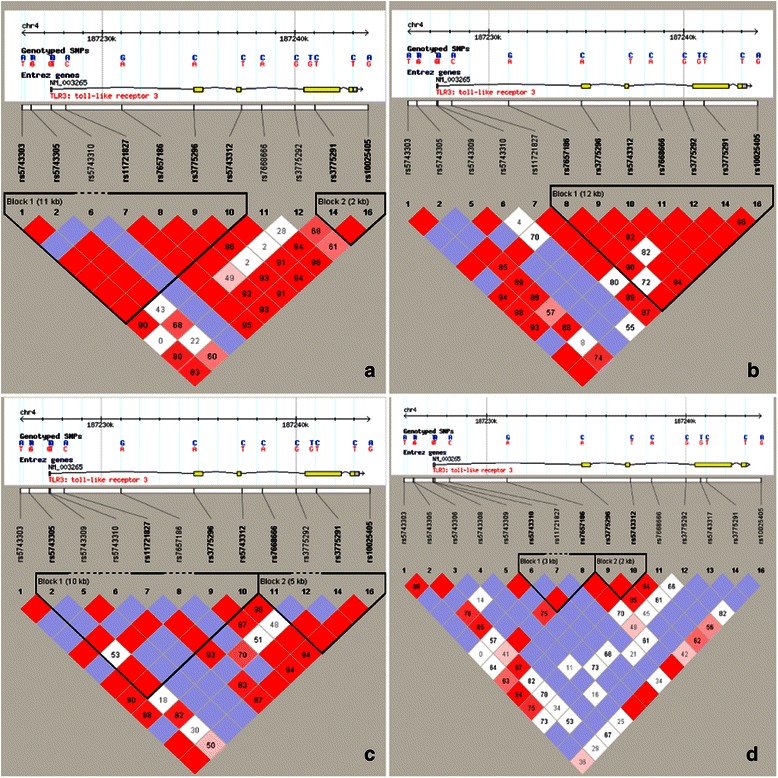
The linkage disequilibrium plot of *TLR3* gene polymorphisms found in individuals of different ethnicities, expanding 20 kb from 5′ to 3′ each (downloaded from the HapMap database). **a**, European; **b**, Chinese Han; **c**, Japanese; **d**, African. Different SNP distributions arise in response to differences in ethnicity. Therefore, differences in ethnicity should be considered when designing the study. Furthermore, some *TLR3* SNPs might be in linkage disequilibrium, suggesting that a haplotype study may be more effective for predicting or screening susceptible populations

rs3775290 is located in exon 4 of the *TLR3* gene and is also a hotspot *TLR3* SNP. In our stratification analyses, *TLR3* rs3775290 was found to be associated with cancer risk in Asian populations. This may be due to the variability in genetic background between Asians and Caucasians (Fig. 2). In the HapMap database, rs3775290 showed a ratio of 0.217:0.783 for the wild-type A:variant G allele in the HapMap-CEU population and of 0.408:0.592 for the A:G allele in the HapMap-CHB and HapMap-JPT populations (http://www.ncbi.nlm.nih.gov/SNP/snp_ref.cgi?rs=3775290). Variations in ethnic backgrounds play an important role in genetic susceptibility, and genetic differences between Asians and Caucasians may be the reason that these different ethnic populations follow different life styles and are thus exposed to different environmental factors [25]. Population groups that are carrying different genotypes or allele frequencies of the rs3775290 polymorphism may show differences in cancer susceptibility [26]. The C to T variant of rs3775290 results in a silent mutation in phenylalanine residue 459. When a SNP leads to a silent mutation, it does not necessarily indicate that the SNP has no impact on protein dynamics. For example, a silent mutation located in an exon might affect interactions between genetic elements and additional molecules, such as metal ions or transcription factors [27].

To date, the rs3775291 polymorphism has been the most investigated SNP in *TLR3*. In the stratified analysis, *TLR3* rs3775291 was also found to be associated with cancer risk in Asians in a heterozygote model (Table 5). Furthermore, when examining our findings on a subgroup-by-subgroup basis, we found that the small sample size subgroup showed that rs3775291 was associated with a significantly increased cancer risk in the M2 and M4 models, whereas the hospital-based subgroup showed this association in the M1, M3, and M5 models. These findings may have arisen because the 3 corresponding studies each obtained significant results. It is possible that in the Asian subgroup, the rs3775291 SNP was associated with cancer risks due to differences in genetic and environmental backgrounds between Asians and Caucasians. In the subgroup analyses, we found a significant association in the small sample size subgroup but not in the large sample size subgroup. In the large sample size subgroup, the ORs in the 5 included studies were all approximately 1.0, whereas the ORs in the studies conducted by Li *et al*. [9], Zeljic *et al*. [19], and Moumad *et al.* [21] indicated a significant association in the small sample size subgroup. The same was true for the hospital-based analysis. Reliable results could be obtained in these cases based on the high quality of the studies designed to explore the real associations of *TLR3* SNPs with cancer risk. Differences in patients’ genetic or environmental backgrounds might certainly be a common mechanism behind the conclusions of our stratification analysis. Differences in how studies were controlled or documented might also provide an explanation for these conclusions. For example, the studies conducted by Li *et al*. [9] and Moumad *et al*. [21] were well controlled, which might explain why positive results were obtained following the analysis of the small sample size subgroup. Furthermore, rs3775291 is located in exon 4, and the G to A variant results in the change of a leucine to phenylalanine at residue 412, which might provide a mechanistic explanation for the effects of this SNP.

Similar to rs5743312, rs3775292 is an intronic polymorphism that has also been investigated in detail, as its location in intron 3 is near rs3775290 and rs3775291. Thus, this polymorphism might be associated with variability in alternative splicing and further associated with the linkage disequilibrium between rs3775290 and rs3775291 [24]. Two of the included studies showed no association of these SNPs with cancer risk, whereas one showed an association. However, our integrated meta-analysis results did not find any association between the rs3775292 SNP and cancer risk. Additional studies will be required to confirm these results.

Our meta-analysis had several limitations. First, only studies that were published in English or Chinese were included in our analysis, thereby creating potential publication bias. Second, the pooled sample size was relatively limited and therefore could support only preliminary evaluations of the association between various *TLR3* polymorphisms and the incidence of various types of cancer. Additionally, we were not always able to obtain original data from the published literature, such as the age and sex of the patients, or the environmental factors that might have affected the hosts. Thus, we used unadjusted information, whereas a more precise analysis could be conducted if detailed information on the original data were available. Therefore, additional studies are required to improve the reliability of these results.

## Conclusion

In summary, this meta-analysis indicated that the variant allele of *TLR3* rs5743312 is potentially associated with increased cancer risks both in the whole collection of studies and in the large sample size subgroup. In the stratified analysis, the variant genotype of the *TLR3* rs3775290 polymorphism was associated with an increased cancer risk in the Asian subgroup. *TLR3* rs3775291 was also associated with an increased cancer risk in the Asian, hospital-based source of controls, and small sample size subgroups. No association was found between *TLR3* rs3775292 and cancer risk. Additional well-designed, large-scale, and functional studies on *TLR3* SNPs are required to confirm our findings.
